# Supplementary material for: A UMLS-based spell checker for natural language processing in vaccine safety
Source: BMC Med Inform Decis Mak. 2007 Feb 12;7:3. doi: 10.1186/1472-6947-7-3 (PMC1805499; doi:10.1186/1472-6947-7-3)
Supplement: Additional file 13 — RAP application directory. Sets up directory for RAP (RDF application for PHP) source code [file 1472-6947-7-3-S13.gz › rap/doc/tutorial/database_persistence.htm]

Tutorial: Database Persistence


### RDF API for PHP V0.9.1

# Tutorial: Database Persistence

This turorial is part of the RAP - Rdf API for PHP documentation.

  

Radoslaw Oldakowski <radol@gmx.de>
  
October 2004

In this tutorial I will demonstrate how to use the new developed
database backend within RAP by giving some examples of setting the
database connection, as well as creating, retrieving, and manipulating
persistent models.

### Introduction

The database backend for RAP V0.9.1 consists of two classes `DbStore`
and `DbModel`. The former is used to set the database
connection as well as create and retrieve persistent models (DbModels).
The latter provides methods for manipulating these models. There is
also a parent class `Model` with some general functions
common for both `MemModel` and `DbModel`.

### First Steps - Setting the Database Connection and Creating Tables

RDF API for PHP V0.9.1 supports a great variety of different databases
(see ADODB supported
databases) . Moreover, RAP allows connecting to multiple databases
at the same time. Every database containing RDF data is represented by
an instance of class `DbStore`. We can create this object
by simply passing all connection parameters to the constructor
function. For example, if we want to use MySQL, we will set the
connection as shown below:

`$mysql_database = ModelFactory::getDbStore('MySQL', 'host',
'db_name', 'user_name', 'password');`

The parameters to be passed represent the database driver, host
name, database name, user name, and password respectively, reflecting
our own connection settings. Similarly, we can connect to other
databases, for instance Oracle:

`$oracle_database = ModelFactory::getDbStore('Oracle', FALSE,
'db_name', 'username', 'password');`

In case that we are using the same type of database all the time, we
can also define the connection parameters as the default setting in
Section `Database` of the file `constants.php`.
For instance, if we use MsAccess via ODBC, with `RDF_DB` as
system DSN, and we do not need any username or password to connect to
our database, we will define the connection constants as follows:

**constants.php**

`//
----------------------------------------------------------------------  
// Database  
//
----------------------------------------------------------------------  
define("ADODB_DB_DRIVER", "ODBC");  
define("ADODB_DB_HOST", "RDF_DB");  
define("ADODB_DB_NAME", "");  
define("ADODB_DB_USER", "");  
define("ADODB_DB_PASSWORD", "");`

Subsequently we can connect to our database using these settings as
default parameters:

`$access_database = ModelFactory::getDbStore();`

When using RAP's database backend for the very first time, we must
additionally create appropriate tables, in order to be able to store
RDF models. RAP provides a very useful method `createTable()`
which will automatically create all tables and indexes needed. We only
have to specify the type of the database we are using:

`$access_database->createTables('MsAccess');  
$mysql_database->createTables('MySQL');`

Unfortunately, currently this method only supports MsAccess and
MySQL. Thus, if we want to use other databases (for instance Oracle, we
have connected to in this tutorial) we will have to create all required
tables and indexes manually by ourselves according to the abstract  database schema provided with the
API documentation.

### Creating and Retrieving DbModels

In RDF API for PHP V0.9.1 every persistent model (DbModel) is given a
unique URI (*modelURI*) to differentiate it from other models.
Thus, when creating a new DbModel or retrieving an existing one, we
have to specify its modelURI. For example, we can create a new, empty
DbModel in the Access database from the previous section by calling the
method `getNewModel()` on the database object and passing
the modelURI as parameter:

`$modelURI =
"http://somewhere.edu/rap-v-06/tutorial/DbModel1";  
$dbModel1 = $access_database->getNewModel($modelURI);`

If we defined the connection parameters in constants.php, we can get an default DbModel without manualy creating an DbStore object before. If the model with the given base URI does exist in the database, it will be opened. If not, it will be created.

`$modelURI = "http://somewhere.edu/rap-v-06/tutorial/DbModel1";  
$dbModel1 = ModelFactory::getDefaultDbModel($modelURI);`

Note that `getNewModel()` will return `FALSE`
if a DbModel with the same modelURI already exists. Therefore, if you
are in doubt, it is highly recommended to use the method `modelExists()`
to check the URI to be assigned, prior to calling `getNewModel()`.
Accordingly, we can modify the above code as follows:

`$modelURI =
"http://somewhere.edu/rap-v-06/tutorial/DbModel1";`

`if ($access_database->modelExists($modelURI))   
   echo "WARNING! DbModel with the same URI: '$modelURI'
already exists";   
else  
   $dbModel1 =
$access_database->getNewModel($modelURI);`

Trying to use an existing modelURI will consequently cause a warning
notice.

It often happens that we not only need to create a completely new
DbModel but moreover want to store an in-memory model or even a
persistent model from another database. For this purpose we can use the
method `putModel()`. To demonstrate this we initially
generate a new MemModel from an RDF document (example1.rdf):

`// Create a new memory model  
$memModel = ModelFactory::getDefaultModel();`

`// Load and parse document  
$memModel->load("example1.rdf");`

and then we pass the object `$memModel` just created,
together with the modelURI we want to assign it, to the method `putModel()`:

`$modelURI =
"http://somewhere.edu/rap-v-06/tutorial/DbModel2";  
$access_database->putModel($memModel, $modelURI);`

To list all models persistently stored in the database RAP provides
the method `listModels()` which returns an array with
modelURI and baseURI of every DbModel. Using this function we can, for
instance, print out the contents of the database created in this
tutorial:

`// get the list with modelURI and baseURI of all
DbModels  
$list = $access_database->listModels();`

`// print out the database contents  
foreach ($list as $model) {  
   echo "modelURI: " .$model['modelURI'] ."<BR>";  
   echo "baseURI : " .$model['baseURI']
."<BR><BR>";  
}`

This will result in the following output:

**Output:**

modelURI: http://somewhere.edu/rap-v-06/tutorial/DbModel1  
baseURI :

modelURI: http://somewhere.edu/rap-v-06/tutorial/DbModel2  
baseURI : http://www.w3.org/Home/Lassila.rdf#

Note that we have not indicated any baseURI for the first model
listed above. We could have done it by passing the baseURI as the
second parameter to the method `getNewModel()`. In the case
of the next model, the baseURI comes from the original MemModel saved
by using the function `putModel()`.

Retrieving a persistent RDF model is also very easy. We simply call
the method `getModel()` on a database object and indicate
the URI of the DbModel to be accessed, as shown below:

`$modelURI =
"http://somewhere.edu/rap-v-06/tutorial/DbModel2";  
$DbModel2 = $access_database->getModel($modelURI);`

`getModel()` returns an object `DbModel` or `FALSE`
if the modelURI was not found.

### Manipulating a DbModel

Once we have created an instance of DbModel, we can manipulate it,
i.e. add, remove, or replace statements, serialize the model into
different formats (plain text, RDF/XML, N3), save it to file or output
in a browser window, compare and combine it with other MemModels or
DbModels, reify, or query it. For this purpose RDF API for PHP allows
us to use the same method names as in the case of manipulating a
MemModel (see tutorial getting started).
Therefore, it is not necessary to repeat them once again. Although the
internal implementation of these methods differs from those of the
class `MemModel`, all the differences are hidden for the
user. Hence, one can expect the same results while manipulating RDF
models regardless of the storage mechanism chosen.

Though, there are two methods specific only for DbModels, namely: `delete()`
removing a persistent model from database and `getMemModel()`
which loads a DbModel (including all statements) into a memory model.
Remember also that the Statement Iterator (`getStatementIterator()`)
is not accessible for instances of DbModel, likewise the indexing
method (`index()`). The latter, because RAP makes use of
database internal indexing capabilities. All the other features
described in the introductory tutorial getting
started are common for both classes `MemModel` and `DbModel`.
